# Metabonomic Profile and Signaling Pathway Prediction of Depression-Associated Suicidal Behavior

**DOI:** 10.3389/fpsyt.2020.00269

**Published:** 2020-04-16

**Authors:** Song Liu

**Affiliations:** ^1^ Department of General Surgery, Nanjing Drum Tower Hospital, Nanjing, China; ^2^ Medical School of Nanjing University, Nanjing, China

**Keywords:** metabolites, depression, signalling, metabonomic profile, suicidal

## Abstract

Suicide is the most severe consequence of depression which has become a leading cause of disability and a global disease burden. Recent evidence indicates a central role of small molecules in the pathogenesis of depression and associated suicidal behaviors. However, there lacks a systemic exploration of small molecules in the development of depression-associated suicide, and it remains unclear how they affect an individual’s behavior. In order to compare the metabonomic profiles between drug-naïve patients with depression-associated suicidal behaviors and healthy individuals, we conducted a systemic database search for studies of metabolic characteristics in depression-associated suicidal behavior. Manual data curation and statistical analysis and integration were performed in Excel. We further performed an enrichment analysis of signaling pathway prediction using the Reactome Pathway Analysis tool. We have identified 17 metabolites that expressed differently between drug-naïve patients with depression-associated suicidal behaviors and healthy controls. We have integrated these metabolites into biological signaling pathways and provided a visualized signaling network in depressed suicidal patients. We have revealed that “transport of small molecules”, “disease”, “metabolism” and “metabolism of proteins” were the most relevant signaling sections, among which “transport of inorganic cations/anions and amino acids/oligopeptides”, “SLC-mediated transmembrane transport”, and “metabolism of amino acids and derivatives” should be further studied to elucidate their potential pathogenic mechanism in the development of depression and associated suicidal behavior. In conclusion, our findings of these 17 metabolites and associated signaling pathways could provide an insight into the molecular pathogenesis of depression-associated suicidal behavior and potential targets for new drug inventions.

## Introduction

Depression is a common mental disease that affects approximately 350 million individuals globally (1). The incidence varies between continents, among which Asia ranks second worldwide with a pooled incidence of 13.5~20.4% in the recent two decades (2). Depression has become one of leading causes of disability and global disease burdens (3).

Suicide is the most severe consequence of depression (4), of which the incidence is estimated around 2~9% among depressed patients (5). However, some consider the number to be far underestimated because suicidal ideation, plans or attempts have not been fully included (6). Notably, the prevalence of depression in physicians (7, 8) and the risk of depression-associated suicide in physicians (9–11) are considerably higher than those in the ordinary population.

A series of studies have suggested a crosstalk between inflammation-associated signaling pathways and the neuroimmune system in the brain that drives the development of depression and behaviors such as suicide (12, 13). Disruption in peripheral and central immune systems appears to contribute to a vulnerability to mental disorders (14).

Emerging evidence strongly indicates a key role of small molecules, especially metabolites, in the pathogenesis of depression (15–18). However, it remains unclear how small molecules act on the brain and affect an individual’s behavior, which is essential for improving the sensitivity of current antidepressant drugs and exploring the diagnostic and therapeutic potential of targeting small molecules for future management of depression.

The utilization of metabonomic approaches can provide high-throughput data that enables simultaneous measurement of numerous small molecules in one specific sample. Considerable studies using the metabonomic technique have been performed to find differentially expressed small molecules between depressed patients versus healthy population, drug-naïve versus drug-treated depressed patients, and drug-sensitive versus drug-resistant depressed patients.

Nevertheless, there appears to be a lack of a systemic summary of significant metabolites in depression, especially in depression-associated suicidal behavior. In the paper, we aim to systemically summarize current evidences of metabolic characterization in depression-associated suicidal behavior, including the name of metabolite, source of tissue type and expression pattern of metabolites. Furthermore, we sought to perform an enrichment analysis to integrate all significant metabolic molecules into biological signaling pathways. We expect that the identification of metabolites and the prediction of signaling pathways can help to elucidate the role of small molecules in the development of depression and associated suicide.

## Methods

### Literature Search Strategy

We conducted a systemic literature search of studies for metabolic characteristics in depression-associated suicidal behavior within the following databases: PubMed/Medline, Embase, Cochrane Library and regional Chinese databases including CNKI, VIP and Wanfang. For instance, the search strategy in PubMed was as follows: ((((((((depression[MeSH Terms]) AND suicide[MeSH Terms]) AND small molecular libraries[MeSH Terms]) AND brain disease, metabolic[MeSH Terms]) AND brain disorder, metabolic[MeSH Terms]) AND depress*[Title/Abstract]) AND suicid*[Title/Abstract]) AND metabo*[Title/Abstract]) AND molecu*[Title/Abstract].

The inclusion criteria were (1) clinical studies involving human samples, (2) studies comparing metabolites between drug-naïve depressed suicide attempters and healthy controls. We accepted studies that utilized nuclear magnetic resonance (NMR), magnetic resonance spectroscopy (MRS), gas chromatography-mass spectrometry (GC-MS), liquid chromatography-mass spectrometry (LC-MS) and other NMR- or MS- based metabolomics technologies.

The exclusion criteria were (1) non-human studies such as animal, primary cell or cell line experiment, (2) other detection technology than those described above, (3) other types of reports such as editorial, case report, commentary, review and study protocol, (4) other language studies than English or Chinese, (5) single-arm studies (non-comparative studies), absence of healthy individuals as control group, irrelevant epidemiology or duplicate studies.

### Data Extraction

The following data were manually extracted from the original publication: name of metabolite, source of tissue type (including both peripheral and central system) and change of expression (either up- or down- regulated).

Distribution of tissue type was generated and presented as a pie chart by Excel (Microsoft, ver. 2016). Manual data curation, statistical analysis and integration of the metabolite map were performed in Excel as well. All significant metabolites were shown in this map as white circles and were connected with their relevant tissue type, shown as black circles. The weight of lines was positively correlated with the number of supporting references. The size of the black circles was positively correlated with the frequency of having been studied in the literature.

### Enrichment Analysis of Signaling Pathways

All significant metabolites were labelled using KEGG C-number and brought into the Reactome Pathway Analysis tool for enrichment of signaling pathways (19, 20). All non-human identifiers were converted to the human equivalents.

### Ethics

As this was a study of literature review and analysis, patient content was not required by the Ethics Committee at Nanjing Drum Tower Hospital.

## Results

After literature screening and eligibility identification, a total of 31 original studies were eventually enrolled into data analysis (21–51) (**Table 1**). Overall, 17 metabolites were found differentially expressed between depressed suicide attempters and healthy controls. They are acetic acid, acetone, cholesterol, d-glucose, glycine, L-alanine, LDL (low-density lipoprotein), L-glutamine, L-lactic acid, L-valine, putrescine, pyruvic acid, quinolinic acid, spermidine, taurine, unsaturated lipid, and VLDL (very low-density lipoprotein).

**Table 1:** Summary of metabolites in depression-associated suicide attempters.

Metabolite	Tissue Type	Expression	Reference
Acetic acid	Urine	Up	21, 22
	Urine	Down	23
	Plasma	Down	24
Acetone	Urine	Up	25
	Urine	Down	22, 26
	Plasma	Up	24
Cholesterol	Plasma	Down	24, 27
D-glucose	Urine	Up	28
	Plasma	Up	24
	Plasma	Down	29
	Serum	Down	30
Glycine	Urine	Up	26, 31
	Plasma	Up	27
	Plasma	Down	24, 29, 32
L-alanine	Urine	Up	22, 26, 31, 33
	Urine	Down	21
	Plasma	Up	27, 34, 35, 36
	Plasma	Down	24, 29, 32
LDL	Plasma	Up	24, 32
L-glutamine	Plasma	Up	29
	Plasma	Down	24, 32, 37
	Serum	Up	30
	Cerebrospinal fluid	Up	38
	Subcortical nuclei	Down	39
	Putamen	Up	40
L-lactic acid	Urine	Up	22, 26, 41
	Urine	Down	21, 23, 42
	Plasma	Up	29
	Plasma	Down	24
	Cerebrospinal fluid	Up	43, 44
	Pregenual anterior cingulate cortex	Up	45
L-valine	Urine	Up	23
	Urine	Down	22
	Plasma	Up	36
	Plasma	Down	24, 29, 32
	Peripheral blood mononuclear cell	Down	46
Putrescine	Frontal cortex	Up	47
Pyruvic acid	Plasma	Down	24, 32, 48
Quinolinic acid	Urine	Up	23
	Urine	Down	22, 28
	Cerebrospinal fluid	Up	49
	Ventrolateral prefrontal cortex	Down	50
Spermidine	Frontal cortex	Up	47
Taurine	Urine	Up	26, 31
	Urine	Down	21, 23
	Plasma	Up	24
	Plasma	Down	51
Unsaturated lipid	Plasma	Up	32
	Plasma	Down	24
VLDL	Urine	Up	25
	Urine	Down	22
	Plasma	Up	24, 32

We summarized the distribution of involved tissue types (**Supplementary Figure 1**). The majority of studies (88%) investigated peripheral tissues including plasma (46%), urine (38%), serum (3%) and peripheral blood mononuclear cells (1%). The remaining 12% studies measured metabolite expression in the central system including cerebrospinal fluid (5%), frontal cortex (3%), subcortical nuclei (1%), putamen (1%), pregenual anterior cingular cortex (1%) and ventrolateral prefrontal cortex (1%). The inaccessibility of central system tissues hampers the investigation towards metabolite expression in the central system.

L-alanine was differentially expressed in peripheral tissues between depression suicide attempters and controls according to 12 references, in which a controversial expression pattern was observed (4 references supported up-regulation, while 1 supported down-regulation in urine; 4 references supported up-regulation, while 3 supported down-regulation in plasma).

L-lactic acid was found in 11 references with different expression levels between diseased patients and healthy controls. In the central system, including cerebrospinal fluid and the pregenual anterior cingulate cortex, L-lactic acid was up-regulated in depressed suicide attempters. In the peripheral system, including urine and plasma, inconsistent expression patterns were found according to respective studies.

L-glutamine was differentially expressed in both peripheral and central tissues between diseases and controls according to 8 references that measured 2 peripheral and 3 central tissues. Similar to L-alanine, these 8 studies failed to reach a consistent result in the L-glutamine expression pattern. L-valine, glycine and taurine were found differentially expressed between depression suicide attempters and healthy controls according to 7, 6 and 6 studies, respectively. Notably, inconsistent expression pattern of these metabolites is observed between studies as well.

**Figure 1:**
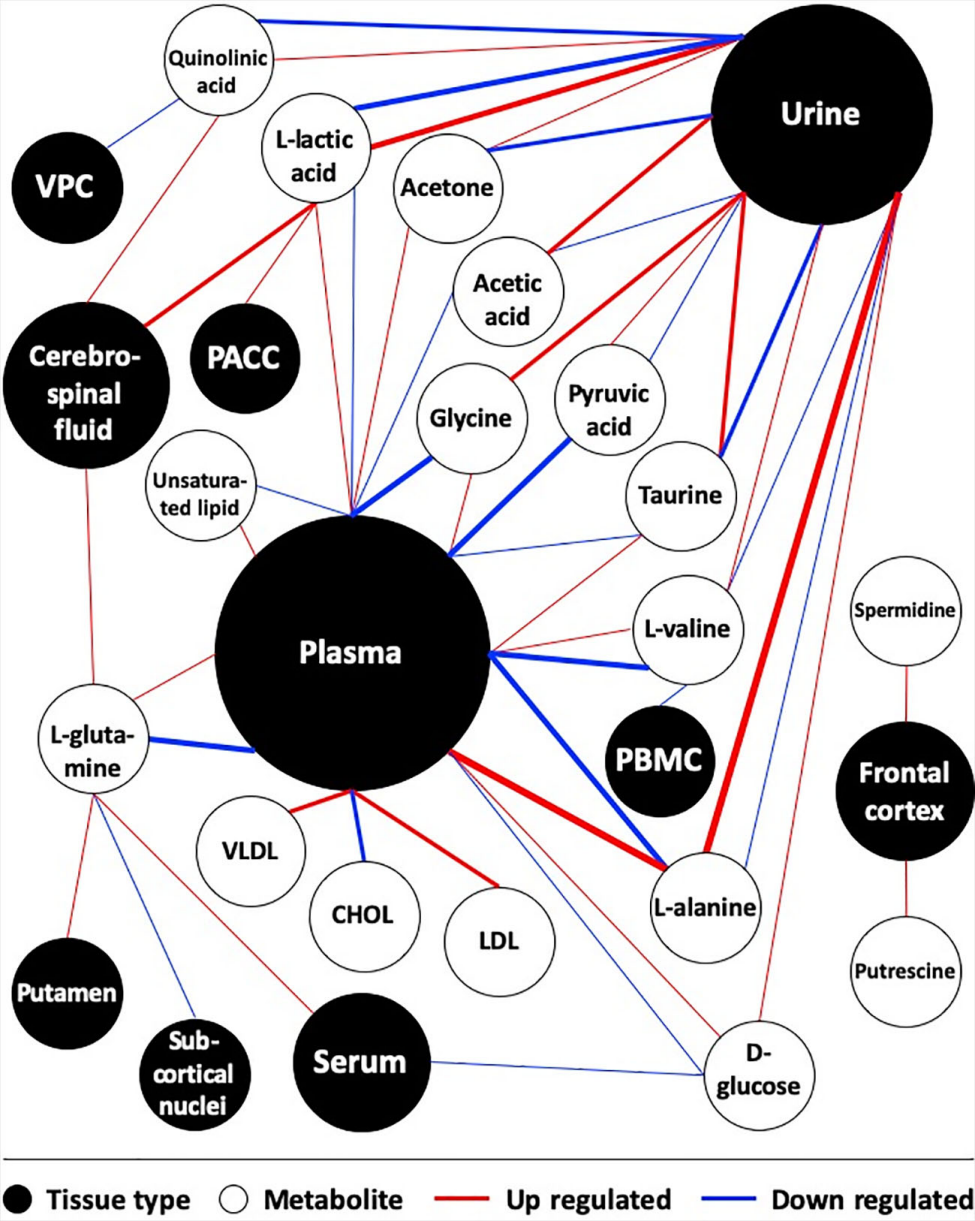
Map of metabolites in depression-associated suicide. Studies comparing metabolites between depressed suicide attempters and healthy controls were included. Weight of lines positively correlates with the number of supporting references. Size of circles positively correlates with the frequency of being studied in the literature. CHOL, Cholesterol; PACC, pregenual anterior cingular cortex; PBMC, peripheral blood mononuclear cell; VPC, Ventrolateral prefrontal cortex.

Multiple studies suggested discrepant expressions of quinolinic acid, acetic acid, acetone and VLDL between diseased patients and controls. However, similar to the above metabolites, their expression patterns were controversial between studies as well. The other metabolites were surveyed by very few references according to which only provisional conclusions could be drawn. We generated a metabolite map to present the expression pattern of all significant metabolites in associated tissues (**Figure 1**).

Next, we utilized the Reactome database to integrate all significant metabolic molecules into biological signaling pathways. Based on reactions being reported by the literature, all metabolites participating in reactions would be created as a network of biological interactions and integrated into signaling pathways.

**Figure 2:**
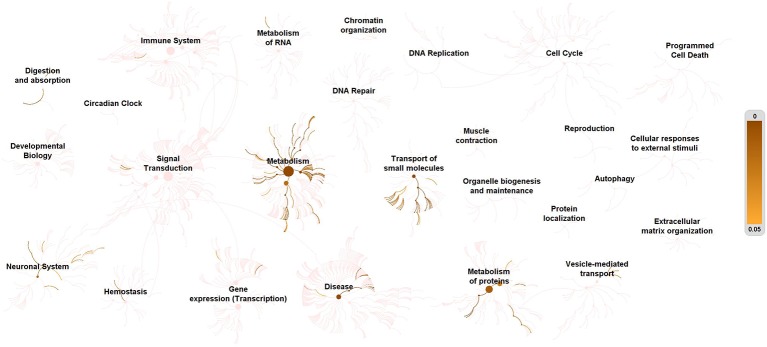
Enrichment of signaling pathways of metabolites in depression-associated suicide. All metabolites in depression-associated suicidal behavior were defined as KEGG C-numbers and brought into Reactome Pathway Analysis. Relevant pathways were highlighted. The right side bar indicates p-value.

**Figure 2** provided a visualization of the signaling network of metabolites involved in depressed suicidal attempters. **Table 2** illustrated the top 20 related signaling pathways, which was divided into 4 sections, including “transport of small molecules”, “disease”, “metabolism”, and “metabolism of proteins”. Among the 20 pathways, “transport of inorganic cations/anions and amino acids/oligopeptides” was assumed as the most relevant signaling pathway which had included 8 metabolites and demonstrated the most significant *p*-value. Besides, “SLC-mediated transmembrane transport” and “metabolism of amino acids and derivatives” contained the largest number of associated molecules, each of which had included 9 metabolites.

**Table 2:** Top 20 signaling pathways that differentially-expressed metabolites participated in depression-associated suicide.

Pathway name	Metabolites involved	*p*-value
*Transport of small molecules*		
Transport of inorganic cations/anions and amino acids/oligopeptides	8	1.1×10^-8^
Transport of bile salts and organic acids, metal ions and amine compounds	8	1.5×10^-7^
SLC-mediated transmembrane transport	9	3.3×10^-6^
Na+/Cl- dependent neurotransmitter transporters	5	5.5×10^-6^
Amino acid transport across the plasma membrane	5	8.6×10^-6^
Organic anion transporters	3	2.9×10^-4^
Proton-coupled neutral amino acid transporters	2	5.7×10^-4^
*Disease*		
Defective SLC6A19 causes Hartnup disorder (HND)	4	4.5×10^-6^
Disorders of transmembrane transporters	7	1.2×10^-5^
SLC transporter disorders	6	5.7×10^-5^
Variant SLC6A14 may confer susceptibility towards obesity	3	2.9×10^-4^
Defective SLC16A1 causes symptomatic deficiency in lactate transport (SDLT)	2	8.9×10^-4^
*Metabolism*		
Metabolism of water-soluble vitamins and cofactors	7	6.3×10^-5^
Metabolism of vitamins and cofactors	8	8.0×10^-5^
Nicotinate metabolism	5	8.5×10^-5^
Metabolism of amino acids and derivatives	9	2.2×10^-4^
Alanine metabolism	2	8.9×10^-4^
*Metabolism of proteins*		
Mitochondrial tRNA aminoacylation	4	6.7×10^-5^
Cytosolic tRNA aminoacylation	4	6.7×10^-5^
Translation	4	2.1×10^-4^

## Discussion

An initial purpose of this study was to identify all metabolites that expressed significantly different between drug-naïve depression patients with suicidal behavior and healthy population, according to current metabonomic studies. A subsequent purpose was to predict relevant signaling pathways that these metabolites theoretically participated in. In summary, we have successfully identified 17 metabolites that expressed differently between drug-naïve patients with depression-associated suicidal behaviors and healthy controls. The expression pattern of metabolites was inconsistent and even contrasted between studies. Peripheral tissues, especially plasma and urine, were extensively investigated in contrast to central system tissues that were difficult to obtain. We have integrated these 17 metabolites into biological signaling pathways and provided a visualized signaling network of metabolites involved in depressed suicidal patients. We have revealed that “transport of small molecules”, “disease”, “metabolism” and “metabolism of proteins” were the most relevant signaling sections, among which “transport of inorganic cations/anions and amino acids/oligopeptides”, “SLC-mediated transmembrane transport”, and “metabolism of amino acids and derivatives” should be further studied to elucidate their potential pathogenic mechanism in the development of depression and associated suicidal behavior.

SLCs (solute carriers) are the largest cluster of transmembrane transporters in charge of various substances exchange, including cations/anions and nutrients as well as transfer of drugs across the blood-brain barrier (52, 53). The function of SLCs is essential for the homeostasis of the brain, since they participate in energy and glutamate metabolism, neurotransmitter release, blood-brain barrier, *etc.* (54). Notably, both mice and human studies emphasized the importance of SLC6A15/v7-3 in the development of depression, of which the mechanism is associated with a neuronal circuits alteration that raises susceptibility to depression (55, 56).

The association between SLCs and a genetic predisposition of suicide was reported by Ernet *et al.* in 2009. They identified that SLC38A1 (also known as SNAT1) was significantly decreased in the brain of suicidal patients (57). A series of SLCs control multiple steps of GABA-glutamate metabolism, which is critical for brain homeostasis as well (58, 59). Dysfunction of SLCs or a disturbed GABA-glutamate cycle is related to the structural, functional and neurochemical impairment in neurons that are observed in patients with major depression (60). Whole genome analysis in brains of depression-associated suicidal victims found alteration of glutamate and GABA receptor genes in this condition (61), which was confirmed by subsequent studies (62–64). Nevertheless, the control subjects in these studies had not suffered from depression or committed suicide. To better clarify whether these gene alterations were caused by depression or suicide *per se*, Zhao *et al.* conducted a comparative study including major depression-committed suicide, major depression/non-suicide and matched healthy controls. They observed that the glutate-glutamine cycle was significantly changed in depressed suicide patients (65).

The pathophysiological role of liver in the development of depression was suggested by previous studies. Bile salt sulfotransferase 1 (SULT2A1) was associated with inflammation response, immune regulation and lipid metabolism in patients with major depressive disorder (66). Organic acid transportation that contributes to depression has been found, and a blocking therapy of organic cation transporter has shown antidepressant efficacy in patients with depression (67). In addition, several transporters of mental ions such as copper and zinc have been confirmed to promote the development of depression (68, 69).

Current diagnosis of depression is based on behavioral symptoms, and traditional anti-depressive drugs are ineffective for a considerable portion of depression patients. Although suicide is the most severe form of reaction to depression, the majority of depressed patients never attempt or commit suicide, implying a pathogenic mechanism of suicidal behavior distinct from other forms of reaction. Recent evidence is emerging to indicate a central role of small molecules in the development of depression. Understanding the pathogenic mechanism of small molecules in depression-associated suicide could promote the prevention, diagnosis and treatment of depressed patients at risk of suicide.

In conclusion, we report here a total of 17 metabolites that express differently between drug-naïve patients with depression-associated suicidal behaviors and healthy individuals. We also predict several signaling pathways that could participate in the development of depression and associated suicide. Our findings could provide an insight into the molecular pathogenesis of depression and potential targets for new drug inventions.

## Data Availability Statement

The datasets generated during and analyzed during the current study are available from the corresponding author on reasonable request.

## Ethics Statement

The studies involving human participants were reviewed and approved by Ethics Committee at Nanjing Drum Tower Hospital. Written informed consent for participation was not required for this study in accordance with the national legislation and the institutional requirements.

## Author Contributions

SL performed data analysis and drafted the manuscript. All members of the Class 2005 of Medical School of Nanjing University designed the research, collected the data, reviewed and approved the manuscript for submission.

## Consortium

***Name list of the Class 2005 of Medical School of Nanjing University (Chinese name is shown in parentheses):*** CHEN Jinyun (陈锦云) ^2^, CHEN Kang (陈康) ^2^, CHEN Liang (陈亮) ^2^, CUI Shiyun (崔诗允) ^2^, DAI Xinjue (戴欣珏) ^2^, DING Chao (丁超) ^2^, DUAN Guihua (段桂华) ^2^, GAO Yibing (高逸冰) ^2^, GENG Linyu (耿林玉) ^2^, GOU Liming (苟黎明) ^2^, GU Xiaoling (顾晓凌) ^2^, GUO Wei (郭伟) ^2^, GUO Zhen (郭振) ^2^, HAN Lijuan (韩丽娟) ^2^, JIANG Kuan (蒋宽) ^2^, LEI Peng (雷鹏) ^2^, LI Jianhua (李建华) ^2^, LI Liyun (李励芸) ^2^, LI Ran (李苒) ^2^, LIANG Fengjing (梁凤晶) ^2^, LING Zhonghua (凌中华) ^2^, LIU Bing (刘兵) ^2^, LIU Song (刘颂) ^2^, LIU Shu (刘澍) ^2^, LIU Yuan (柳元) ^2^, MA Chunyan (马春燕) ^2^, MA Hucheng (马虎成) ^2^, NIU Yuxiang (牛宇翔) ^2^, OU Wei (欧伟) ^2^, QIN Jizheng (覃基政) ^2^, SHA Shifu (沙士甫) ^2^ *DECEASED*, SHEN Juanhong (沈娟红) ^2^, SUN Jing (孙晶) ^2^, SUN Qing (孙青) ^2^, WANG Fei (汪飞) ^2^, WANG Kongcheng (王孔成) ^2^, WANG Xinqiang (王鑫强) ^2^, WANG Xinyi (王昕怡) ^2^, WEN Juan (闻娟) ^2^, WU Buyun (邬步云) ^2^, WU Han (吴韩) ^2^, WU Li (吴丽) ^2^, WU Puyuan (吴浦嫄) ^2^, WU Xiaodong (吴晓东) ^2^, XIA Haiyan (夏海燕) ^2^, XIA Qiuyuan (夏秋媛) ^2^, XIE Zhigang (谢志刚) ^2^, XU Weiwei (徐维玮) ^2^, YAN Jizhang (延吉璋) ^2^, YANG Nan (杨楠) ^2^, YANG Yan (羊妍) ^2^, YAO Danhua (姚丹华) ^2^, Ye Xie (叶叶) ^2^, ZHANG Haifeng (张海峰) ^2^, ZHANG Luyao (张璐瑶) ^2^, ZHANG Xiaolei (张小磊) ^2^, ZHANG Yiyan (张艺艳) ^2^, ZHAO Xin (赵鑫) ^2^, ZHENG Yang (郑阳) ^2^, ZHOU Wei (周薇) ^2^, ZHU Genfei (祝根飞) ^2^, ZHU Jianhong (祝剑虹) ^2^.

## Funding

This study is supported by the National Natural Science Foundation of China (SL) (81602103), Natural Science Foundation of Jiangsu Province (SL) (BK20160114), Distinguished Young Scholar Project of Medical Science and Technology Development Foundation of Nanjing Department of Health (SL) (JQX17005), Key Project of Medical Science and Technology Development Foundation of Nanjing Department of Health (SL) (YKK16114), Medical Research Program of Jiangsu Provincial Commission of Health and Family Planning (SL) (Q2017007), and Wu Jieping Medical Foundation (SL) (320.2710.1817).

## Conflict of Interest

The author declares that the research was conducted in the absence of any commercial or financial relationships that could be construed as a potential conflict of interest.

## Supplementary Material

Supplementary Figure 1 Distribution of tissue type in metabolite-associated studies. Studies that compare metabolites between depressed suicide attempters and healthy controls were included. PACC, pregenual anterior cingular cortex; PBMC, peripheral blood mononuclear cell; VPC, Ventrolateral prefrontal cortex.
