# Supplementary figures and images for: Metabonomic Profile and Signaling Pathway Prediction of Depression-Associated Suicidal Behavior
Source: Front Psychiatry. 2020 Apr 16;11:269. doi: 10.3389/fpsyt.2020.00269 (PMC7177018; doi:10.3389/fpsyt.2020.00269)

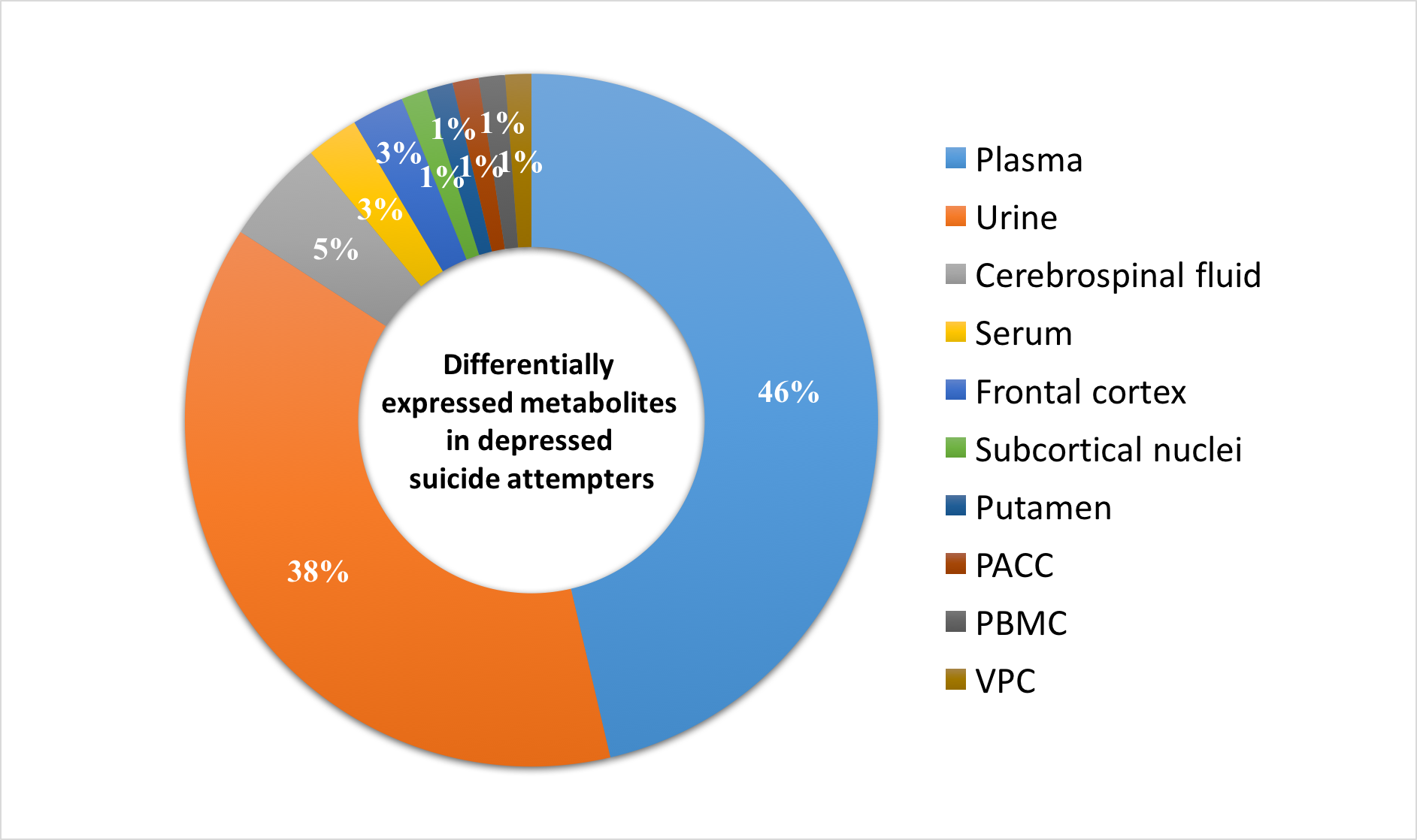

Supplement: Supplementary Figure 1 — Distribution of tissue type in metabolite-associated studies. Studies that compare metabolites between depressed suicide attempters and healthy controls were included. PACC, pregenual anterior cingular cortex; PBMC, peripheral blood mononuclear cell; VPC, Ventrolateral prefrontal cortex. [file Image_1.tiff]
